# Effect of BioZorb® surgical marker placement on post-operative radiation boost target volume

**DOI:** 10.1007/s13566-017-0339-y

**Published:** 2018-01-03

**Authors:** Naomi Wiens, Lisa Torp, Brynn Wolff, Yijin Wert, Katherine Barton, David Weksberg, Joella Wilson-Dagar

**Affiliations:** UPMC Pinnacle Cancer Institute, 4300 Londonderry Road, Harrisburg, PA 17109 USA

**Keywords:** Bioabsorbable surgical marker, Breast-conserving therapy, Boost radiation, Radiation reduction, Oncoplastic

## Abstract

**Objective:**

BioZorb® is a tumor bed marker placed during partial mastectomy for targeted post-operative radiation. This study was designed to evaluate BioZorb® effect on radiation boost clinical target volume (CTV), planning target volume (PTV), median dose to ipsilateral lung (Gy), and heart irradiation in left-sided cancers.

**Methods:**

Data was collected via a retrospective cohort study with two study arms: BioZorb® intra-operative placement versus no BioZorb® placement. Patients were stratified by BMI, age, tumor laterality and volume, and cancer stage. Mean, standard deviation, median, range of cubic centimeters of clinical and planning target volume, cardiac dose in left-sided cancers, ipsilateral lung dose, and volume of ipsilateral lung receiving 20 Gy were reported.

**Results:**

Of 143 patients, median CTV (cm^3^) was 8.7 and 14.2 (*P* = 0.0048), median PTV (cm^3^) was 53.2 and 79.6 (*P* = 0.0010), median ipsilateral lung Gy was 7.53 and 6.74 (*P* = 0.0099) and volume (cc) of ipsilateral radiation lung at 20 Gy was 13.4 and 12 (*P* = 0.008), and median heart Gy in left-sided cancers was 2.01 and 2.21 (*P* = 0.9952) in BioZorb® and non-BioZorb® arms, respectively. Patients with BMIs of 25–30 had CTV medians of 7.8 and 11.1 in BioZorb® and non-BioZorb® arms, respectively (*P* = 0.0293).

**Conclusion:**

The BioZorb® arm showed statistically significant reductions in CTV and PTV but not ipsilateral lung or heart irradiation.

## Introduction

Breast-conserving therapy with post-operative radiation is well established and often the preferred procedure for many breast cancer patients with early-stage disease. BioZorb® (Focal Therapeutics, Aliso Viejo, CA, USA) is a 3D, six-clip helical or low-profile surgical marker that is placed in the tumor bed during partial mastectomy and targeted post-operatively with radiation in breast-conserving therapy as demonstrated by Simpson J and Benjamin B at the European Society for Radiotherapy and Oncology Annual Forum, Geneva, Switzerland, in 2013. Traditionally, surgical scar site, intra-operative clip placement, or the presence of seroma have been used to identify the post-operative boost site of radiation. These targeting methods have been shown to vary among radiation oncologists when delineating surgical excision site [1]. Oncoplastic surgical techniques are also being widely implemented as surgeons gain experience in hidden scar breast surgery. This often requires making incisions that are distant from the tumor cavity site for cosmetically appealing outcomes, making post-operative radiation boost dose delivery targeting scars inconsistent or imprecise [2]. Traditional surgical clips placed around the tumor bed have also been known to migrate without oncoplastic techniques, and target volumes are often expanded to include migrated clips [3].

With the use of BioZorb®, the boost dose may be targeted around the surgical marker. The device is typically fixated into the tumor cavity by the surgeon with a four-point fixation using monofilament absorbable sutures. This ensures no clip migration because the titanium clips are incorporated into the marker being fixated into the excision cavity. This bioabsorbable polymer marker was developed both to help identify a surgical excision site and for potentially improved cosmetic outcome, as it fills space to improve breast contour following lumpectomy [4]. The purpose of this study is to determine if using this marker results in smaller boost treatment volumes with the same efficacy as traditional tumor bed targeting using clips, surgical scar, and seroma.

Limited research has been done on whether or not radiation target volumes change with BioZorb® placement and no data has been published on recurrence rates using smaller planning target volumes (PTV). Early data supports that the tumor bed volume of radiation may be decreased in patients who receive the BioZorb® marker compared with traditional lumpectomy patients, as presented by Kara Leonard, MD, at the 2017 American Society of Breast Surgeons (ASBrS) Annual Meeting. More research investigating this topic is needed. Data on secondary organ radiation, i.e., to the ipsilateral lung or heart in left-sided cancers, has yet to be explored.

Whole breast irradiation with hypofractionated boost doses has been shown to improve local recurrence; however, radiation has well-established side effects including tissue ischemia and fibrosis [5]. This study hypothesized that BioZorb® implantation reduces the radiation boost clinical target volume and PTV. Secondary analysis included the mean dose (Gy) to the ipsilateral lung, volume (cc) of the ipsilateral lung receiving 20 Gy, and mean dose to the heart in left-sided breast cancers.

## Methods

Data was collected via a retrospective cohort study. Data collection was obtained from UPMC Pinnacle Cancer Institute, a nationally accredited breast care center and American College of Radiology-accredited community cancer center in Harrisburg, Pennsylvania, from 2015 to 2017. Patients were stratified based on BMI, age, tumor laterality and size, and breast cancer stage. The traditional study arm did not receive intra-operative BioZorb® markers and the other arm had the BioZorb® placed. In both study arms, the surgeons implemented modest oncoplastic techniques with tissue rearrangement and flap creation for hidden scar surgery. Inclusion criteria are patients who underwent lumpectomy with adjuvant whole breast radiation with either standard fractionation (50 Gy in 25 fractions) or a hypofractionated (40–42.4 Gy in 15–16 daily fractions) regimen, followed by a 10–16-Gy boost in 4–8 fractions.

Contouring and treatment delivery was performed before the study was conducted. This was done by three radiation oncologists working with three breast surgeons at a single institution with the exception of four patients in the traditional arm who had surgery at an outside institution. All plans were reviewed and approved at weekly chart rounds. No additional contouring was performed for the study. CTV in the traditional arm was determined by using the operative report, pre-treatment imaging, surgical clips, and seroma. The lumpectomy scar was not routinely included in the CTV in either arm due to hidden scar techniques utilized by the surgeons.

Patients were treated in the supine position using 3D planning (tangents using field-in-field technique). Breast boards and/or Vac-Lok™ were used for immobilization. Deep inspiration breath hold was not used. Boost technique varied based on patient anatomy and location of the boost volume and included two-, three-, and four-field photon plans occasionally with a couch kick, and electron boost. Image guidance was utilized for radiation delivery. BioZorb® was utilized as a fiducial marker for IGRT and patient setup verification for the boost treatments.

Patients who received nodal radiation or post mastectomy radiation were excluded. The mean, standard deviation, median, range of cubic centimeters (cm^3^) of CTV and PTV, median dose (Gy) to the ipsilateral lung, volume (cc) of the ipsilateral lung receiving 20 Gy, and dose to the heart in left-sided breast cancers were reported. The Wilcoxon rank-sum test was used to detect if the two distributions were identical with respect to the median. All analyses were done in SAS version 9.4 (SAS Institute, Cary, NC).

## Results

Data was collected from 143 patients (*n* = 143), 82 in the BioZorb® arm (*n* = 82) and 61 in the traditional, no BioZorb® arm (*n* = 61). There were no statistical differences between the two groups regarding age, BMI, stage, or tumor volume as measured by greatest dimension of largest focus of invasion using final gross and microscopic pathologic evaluation. However, more right-sided breast cancers were seen in the group who had BioZorb® implanted compared to those who did not, 62.2 and 37.7%, respectively (*P* = 0.0037), as shown in Table 1. Median CTV (cm^3^) in the BioZorb® arm was 8.7 and 14.2 in the traditional arm, respectively (*P* = 0.0048). Median PTV (cm^3^) was 53.2 and 79.6 (*P* = 0.0010), median Gy to the ipsilateral lung was 7.53 and 6.74 (*P* = 0.0099) and volume (cc) of radiation to the ipsilateral lung at 20 Gy was 13.4 and 12 (*P* = 0.008), and median heart Gy in left-sided cancers was 2.01 and 2.21 (*P* = 0.9952) in BioZorb® and traditional arms, respectively (Table 2). Higher average BMI correlated with higher mean cubic centimeter of radiation at 20 Gy and mean Gy to the ipsilateral lung in the BioZorb® arm, with respective *P* values of 0.0293 and 0.0021 in patients with BMIs between 25 and 30 and over 30 respectively (Tables 3 and 4). Patients with BMI of 30 or greater had CTV medians of 13.6 and 9.8 in the BioZorb® and traditional arms respectively (*P* = 0.0015, Table 5).

**Table 1:** Patient demographics (age, body mass index (BMI), mean, standard deviation (SD), range, stage, tumor volume as measured by greatest extension of largest focus of invasion (mm), and laterality with corresponding *P* values for BioZorb® and traditional study arms)

	BioZorb®		Traditional		*P* value
Number of patients	82		61		
Age—mean (SD), range	60.1 (10.6)	31–81	60.3 (8.0)	47–83	0.9366
BMI—mean (SD), range	31.7 (6.8)	19–51.2	33.4 (8.4)	21–68	0.1798
BMI by category—no. %					
Underweight BMI < 18	0	0.0%	3	4.9%	0.0754
Normal weight BMI 18–24.9	9	11.0%	9	14.8%	0.5005
Overweight BMI 25–29.9	30	36.6%	15	24.6%	0.1266
Obese BMI 30–34.9	19	23.2%	10	16.4%	0.3188
Severely obese 35–39.9	13	15.9%	12	19.7%	0.5521
Morbidly obese > 40	11	13.4%	12	19.7%	0.3138
Stage					
0	10	12.2%	10	16.4%	0.8069
1A	61	74.4%	42	68.9%	
2	0	0.0%	1	1.6%	
2A	6	7.3%	3	4.9%	
2B	2	2.4%	1	1.6%	
3A	1	1.2%	0	0.0%	
Side					
Left	31	37.8%	38	62.3%	0.0037
Right	51	62.2%	23	37.7%	
Tumor size (mm)—mean (SD), range	11.3 (5.9)	1–32	13.9 (9.6)	4–50	0.0919

**Table 2:** Patient outcomes (clinical target boost volume (CTV), planning target boost volume (PTV), mean, standard deviation (SD), median, and range of radiation values in Gray (Gy), volume (V) of radiation to ipsilateral lung at 20 Gray, with corresponding *P* values for BioZorb® and traditional study arms)

	BioZorb®		Traditional		*P value
Number of patients	82		61		
CTV boost (cm^3^)—mean (SD), median (range)	11.1 (8.4)	8.7 (2.2–54.5)	17.0 (15.8)	14.2 (0.04–104.2)	0.0048
PTV boost (cm^3^)—mean (SD), median (range)	76.3 (91.9)	53.2 (20.5–817.2)	89.7 (45.1)	79.6 (20.84–213.14)	0.0010
Mean Gy heart (left side only)—mean (SD), median (range)	2.24 (103.3)	2.01 (78.9–430.7)	2.18 (67.7)	2.21 (76.5–375.6)	0.9952
Mean Gy ipsilateral lung—mean (SD), median (range)	7.72 (227.7)	7.53 (300.9–1842)	6.58 (213.1)	6.74 (110.6–1192.8)	0.0099
V ipsilateral lung receiving 20 Gy—mean (SD), median (range)	13.7 (5.0)	13.4 (3.3–35)	11.1 (4.4)	12 (0.4–21.7)	0.0080

*Wilcoxon rank-sum test—it tests the null hypothesis that the two distributions are identical with respect to the median

**Table 3:** Volume (V) ipsilateral lung rec 20Gy by BMI category: volume of radiation to ipsilateral lung at 20 Gray (Gy) stratified by body mass index (BMI) category with corresponding mean, standard deviation (SD), median, range, and *P* values)

	BioZorb®		Traditional		*P value
Number of patients	82		61		
BMI < 25—mean (SD), median (range)	8.1 (5.9)	5.2 (3.9–21.8)	17.8 (13.5)	14.7 (2.0–37.9)	0.0817
25 ≤ BMI < 30—mean (SD), median (range)	10.3 (9.4)	7.8 (2.2–54.5)	19.5 (24.7)	11.1 (0.04–104.2)	0.0293
BMI ≥ 30—mean (SD), median (range)	12.2 (8.0)	11.0 (2.2–53.0)	15.6 (11.3)	14.3 (3.7–56.3)	0.1741

*Wilcoxon rank-sum test—it tests the null hypothesis that the two distributions are identical with respect to the median

**Table 4:** Mean Gy ipsilateral lung by BMI category: radiation to ipsilateral lung in Gray (Gy) stratified by body mass index (BMI) category with corresponding mean, standard deviation (SD), median, range, and *P* values

	BioZorb®		Traditional		*P value
Number of patients	82		61		
BMI < 25—mean (SD), median (range)	7.62 (1.8)	7.6 (4.2–11.4)	7.01 (1.3)	7.6 (4.1–8.4)	0.4555
25 ≤ BMI < 30—mean (SD), median (range)	7.69 (2.7)	7.73 (3.0–18.4)	7.34 (2.3)	7.6 (3.1–11.9)	0.9137
BMI ≥ 30—mean (SD), median (range)	7.76 (2.1)	7.39 (3.9–13.4)	6.09 (2.2)	6.1 (1.1–11.0)	0.0021

*Wilcoxon rank sum test—it tests the null hypothesis that the two distributions are identical with respect to the median

**Table 5:** CTV boost (cm^3^) by BMI category: BioZorb® and traditional clinical target boost volumes (cc) by body mass index (BMI) with corresponding mean, standard deviation (SD), median, range, and *P* values

	BioZorb®		Traditional		*P value
Number of patients	82		61		
BMI < 25—mean (SD), median (range)	13.6 (4.1)	13.9 (7.6–22.6)	12.4 (3.1)	13.5 (6.5–15.7)	1.0000
25 ≤ BMI < 30—mean (SD), median (range)	13.6 (5.9)	13.2 (3.3–35)	12.9 (4.6)	14.4 (3.7–21.7)	0.9520
BMI ≥ 30—mean (SD), median (range)	13.7 (4.5)	13.6 (5.3–25.5)	9.9 (4.4)	9.8 (0.4–18.6)	0.0015

*Wilcoxon rank-sum test—it tests the null hypothesis that the two distributions are identical with respect to the median

## Discussion

BioZorb® is an easily visualized surgical marker found to be beneficial in radiation treatment plans [6]. As more surgeons implement oncoplastic techniques into their practice, BioZorb® has become a widely accepted tumor bed cavity marker. All three surgeons in this study consistently used oncoplastic techniques with hidden scar surgery, creating flaps and rearranging tissue after BioZorb® fixation. Recent data supports our hypothesis that BioZorb® implantation reduces radiation boost volumes; however, we are not aware of any study investigating secondary organ radiation doses.

Using the Wilcoxon rank-sum test to analyze results, statistically significant reductions in boost CTV and PTV were seen in the BioZorb® arm, supporting the hypothesis that BioZorb® placement decreases radiation target volumes. No statistical difference was seen in tumor volumes between the two study arms. No difference in heart radiation dose in left-sided breast cancers was noted.

An increased average BMI was shown to correlate with the increased mean cubic centimeter of radiation at 20 Gy and mean Gy to the ipsilateral lung in the BioZorb® arm; however, these results were only statistically significant in BMI values over 25 and 30. This data may depend on whole breast anatomy and shape of underlying rib cage rather than the placement of a particular surgical marker or boost volume delivered.

Interest in stratification by BMI was taken when developing this study because excess fatty tissue was thought to possibly make the boost target less precise. This may be the case, and when the patients were stratified based on BMI, those with over 25 had decreased radiation boost volumes with BioZorb®. This is consistent with the general population of females in America; 70% of females in the USA are overweight or obese [7]. This makes our study very applicable to the average breast cancer patient in the USA.

Targeting methods vary between radiation oncologists. Boost volumes were contoured by three radiation oncologists, rather than a single radiation oncologist. Subsequently, differences in contouring may be a limitation to this study.

Radiation-induced conditions such as fibrosis, ischemia, and neuropathy can be serious and have profoundly negative impacts on patient quality of life [8]. Attempts necessary to reduce the risk of radiation should be made while ensuring treatment remains effective. Actual toxicity and local recurrence rates were not addressed in this study; it cannot be concluded that a smaller boost PTV is clinically beneficial in the population with BioZorb® placement. Smaller clinical volumes may actually increase rates of recurrence. Currently, some research showed partial mastectomies with BioZorb® placement had no recurrent cancer detected on imaging at 36 months follow-up [6]. However, considering this surgical marker is still considerably new, its long-term recurrence rates are unknown. It stands to reason that smaller target volumes may result in less toxicity, but if sacrificed for increased local recurrence rates, the results could be deleterious. Additional investigation into long-term local recurrence, cosmetic satisfaction, actual time until the marker is fully absorbed, and financial justification would also be beneficial.

## Conclusion

This study examines the role of the helical surgical marker BioZorb® in targeting radiotherapy in breast conservation therapy. This is the first study to analyze the dose of nearby organs at risk and demonstrates a reduction in radiation to the breast but increase in ipsilateral lung irradiation. As such, surgeons may choose to place the marker with confidence in accurate targeting, even when oncoplastic techniques are implemented, but caution should be taken to avoid secondary organ irradiation. Additional outcome studies will be needed to assess long-term toxicity, cosmesis, and local recurrence rates associated with the smaller boost PTVs that are achieved with BioZorb®.

Multidisciplinary treatment planning discussions between the breast surgeon, radiation oncologist, and patient remain at the forefront of quality patient care. Overall, the clinical impact of this study is that placement of the BioZorb® marker reduces the radiation boost volumes and thus should be considered in the appropriate clinical setting.
